# Curvature Blindness Illusion

**DOI:** 10.1177/2041669517742178

**Published:** 2017-11-24

**Authors:** Kohske Takahashi

**Affiliations:** School of Psychology, Chukyo University, Nagoya-Shi, Japan

**Keywords:** contours or surfaces, curvature perception, illusion, perception

## Abstract

We report a novel illusion––curvature blindness illusion: a wavy line is perceived as a zigzag line. The following are required for this illusion to occur. First, the luminance contrast polarity of the wavy line against the background is reversed at the turning points. Second, the curvature of the wavy line is somewhat low; the right angle is too steep to be perceived as an illusion. This illusion implies that, in order to perceive a gentle curve, it is necessary to satisfy more conditions––constant contrast polarity––than perceiving an obtuse corner. It is notable that observers exactly “see” an illusory zigzag line against a physically wavy line, rather than have an impaired perception. We propose that the underlying mechanisms for the gentle curve perception and those of obtuse corner perception are competing with each other in an imbalanced way and the percepts of corner might be dominant in the visual system.

Perception of contour and shape is one of basic functions of vision. To this end, visual system processes information in a hierarchical way; first it extracts local orientations, then it integrates the local orientations into intermediate representations of contour, and finally it forms global shape percepts (Loffler, 2008). The intermediate representation of contour would include concavity, convexity, corner angle, curvature, and so forth. Although it is obvious that the physical shape is determined by combination of the local orientations, perceptual shape is susceptible to several factors. Accordingly, as visual illusions demonstrate, percepts are not necessarily veridical. For example, the café wall illusion (Pierce, 1898) demonstrates that parallel horizontal lines are perceived as different angles to each other.

Here, we report a novel illusion––curvature blindness illusion––that will provide novel implications for contour perception, in particular, for the underlying mechanisms of curve and corner perception. Figure 1 demonstrates this illusion. One must see the pairs of wavy lines (i.e., gentle curves) and pairs of zigzag lines (i.e., obtuse corners) on the gray background. Physically, however, all lines are wavy lines with an identical shape; there is no triangular wave and hence there is no corner. One would find that all the lines on the white and black background are wavy. As the effect magnitudes are quite strong, unless one carefully stares at the region that looks like a corner, it is hard to find that all lines are physically wavy. Despite the simplicity and effect magnitudes, to the best of our knowledge, no one has reported about this phenomenon.

**Figure 1.**
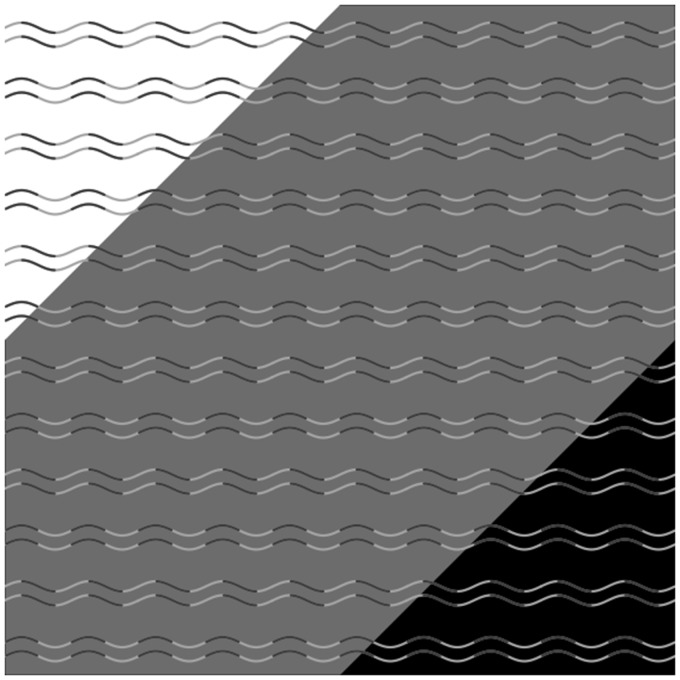
Example of the curvature blindness illusion. Physically, all lines are identical sine wave. However, one would see obtuse corners rather than gentle curves for some sine waves on the gray background.

Psychophysical, physiological, and computational studies have excessively investigated the underlying visual process of curve and corner perception (Loffler, 2008). The basic idea is a hierarchal process, that is, the outputs from local and neighboring orientation filters in V1 are integrated at the later stages, V2, V3, or V4, and the curvature or angle information are extracted as intermediate representations of contours (Dumoulin & Hess, 2007; Ito & Komatsu, 2004; Kramer & Fahle, 1996; Levi & Klein, 2000; Snippe & Koenderink, 1994). The end-stopped cells would also play a role in detecting relatively high curvature (Dobbins, Zucker, & Cynader, 1987, 1989; Skottun, 1998). However, these studies have addressed how the visual system discriminates different curvatures, detects a low curvature from a straight line, discriminates different corner angles, or detects obtuse angles from a straight line; thus, the perception of curve and corner have been studied in a different context.

Accordingly, little is known regarding how the visual system discriminates a curve from a corner and how these two percepts are related to each other (Ito, 2012, may be one exception). As the curvature blindness illusion demonstrates that the low curvatures are perceptually replaced by the obtuse corners, it postulates that the underlying mechanisms of curve and corner perception could not be independent, but rather would interfere or compete with each other. Therefore, disentangling this illusion should provide a unified view of curve and corner perception and also extend the current understandings of contour perception.

In the present study, we conducted three experiments to explore the determining factors and mechanisms underlying curvature blindness. Experiment 1 investigated the effects of the position of color change. Experiment 2 investigated the effects of background luminance. Finally, Experiment 3 investigated which of the two plays a crucial role: color change or line discontinuity.

## Experiment 1

Experiment 1 investigated the effects of the strength of the curvature or angle of wavy and zigzag lines as well as how sensitive to the difference in the position of color change. These investigations would reveal the basic characteristics of the illusion in terms of the spatial configuration.

### Methods

Eight naïve university students participated as paid volunteers after giving written informed consent. All of the participants had normal or corrected-to-normal visual acuity. The study was approved by the Ethics Committee of the University of Tokyo and conducted in accordance with the Declaration of Helsinki.

The experiment was conducted in a quiet and dimmed room. The visual stimuli were presented on a CRT monitor (the refresh rate was 75 Hz, resolution was 1024 × 768 pixels) at a viewing distance of 57 cm. The stimulus presentation and response acquisition were controlled by the Apple Mac Mini and PsychoPy (Peirce, 2007, 2008).

**Figure 2.**
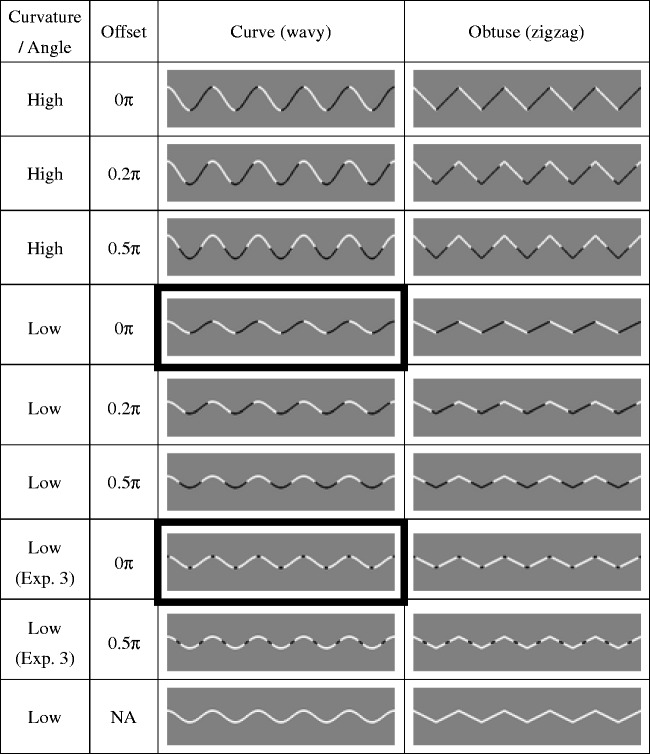
Examples of stimuli presented in Experiments 1 and 3. The cells surrounded by the thick border indicate the conditions inducing the curvature blindness illusion.

The visual stimulus was a triangle wave (zigzag line) or a sinusoidal wave (wavy line) presented on a grey background (18.1 cd/m^2^), as shown in Figure 2. The wavelength of the line was 40 pixel (1.57°) and the horizontal range of the line was 800 pixel (30.7°). We presented two curvature or angle strengths. The range on the vertical axis was 10 pixel (0.39°) and 20 pixel (0.78°) for low and high curvature, respectively; hence, the corner angle was right (90°) and obtuse (126.9°) for the high and low curvature or angle, respectively. The line was painted alternately with black (0.38 cd/m^2^) and white (98.0 cd/m^2^) with an alternation frequency of π. The offset of alternation was manipulated from 0 to 0.5π with 0.1π intervals. The offset 0.5π (e.g., third from top row in Figure 2) indicates that the line color changes in the middle of two turning points, while the offset 0π (e.g., top row in Figure 2) indicates that the line color changes at the turning point. In addition, the line painted with white was also presented (bottom row in Figure 2).

The participant began a trial by pressing a spacebar. A visual stimulus was presented for one frame (13.3 ms) after 500 ms blank screen. The participant’s task was to indicate whether the line was zigzag or wavy by pressing the corresponding key (F or J). After six practical trials, each condition was presented 16 times in a pseudorandom sequence, leading to 448 trials in total (2 shape × 2 angle × 7 offset × 16 repetition).

### Results

**Figure 3.**
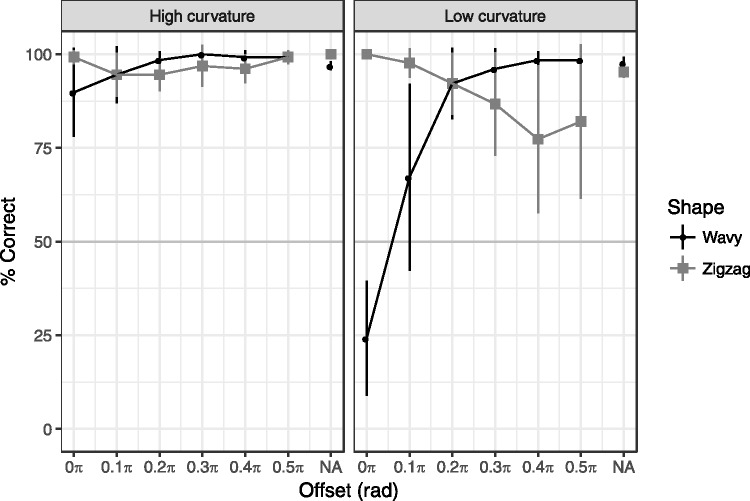
Results of Experiment 1. The chance level was 50% (horizontal gray line). An accuracy rate higher than chance indicates that the percepts were veridical; an accuracy rate lower than chance indicates that the percepts were illusory. Error bars indicate 95% confidential intervals.

Figure 3 shows the results of Experiment 1. A three-way repeated measures ANOVA (with Huynh–Feldt correction) of shape, angle, and offset revealed the significant three-way interaction, *F*(6, 42) = 24.9, *p* < .001, ηp2^ ^= 0.78. Thus, as shown in Figure 3, the effects of offset differed among the conditions. The effect was significant for the low-curvature wavy shape condition, *F*(6, 42) = 45.6, *p* < .001, ηp2^ ^= 0.87. A paired comparison (corrected by Ryans method) revealed that the accuracy of 0π condition was lower than the other conditions as well as that of 0.1π condition was lower than the other (except for 0π) conditions. On the other hand, the effects of offset were not significant in the other conditions—high-curvature wavy shape: *F*(6, 42) = 2.70, *p* = .12, ηp2^ ^= 0.28; low-curvature zigzag shape: *F*(6, 42) = 3.40, *p* = .06, η_p_^2 ^= 0.32; high-curvature zigzag shape: *F*(6, 42) = 2.11, *p* = .10, η_p_^2 ^= 0.23.

We also tested that the accuracy rate was biased to the illusory perception. Consistent with our observation, the results demonstrated that the wavy line with low curvature was misperceived as a zigzag line when the color changed at the turning point (offset = 0π). The accuracy rate was below chance (95% CI = [0.09, 0.40] against the chance level 0.5), which indicates that the participants exactly “see” an illusory zigzag line against a physically wavy line, rather than have an impaired percepts of the line shape.

## Experiment 2

Experiment 2 investigated the effects of the luminance contrast of the line color and the effects of background luminance. These manipulations would reveal which color change of the line per se or the reversed contrast polarity against the background is crucial for the illusion.

### Methods

**Figure 4.**
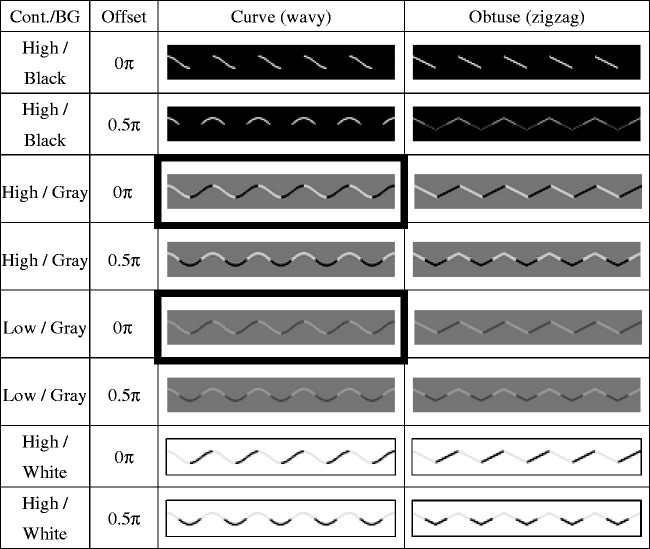
Examples of stimuli presented in Experiment 2. The cells filled with light gray indicate the conditions inducing the curvature blindness illusion.

Eight naïve university students different from those participating in Experiment 1 were recruited. The methods were identical with Experiment 1, except for the following. Figure 4 shows some examples of the visual stimuli. In Experiment 2, only the low curvature or angle stimuli were presented. The offset of the color change was either 0.0, 0.17, 0.33, 0.50π, or no color change (white line). The background was either black (pixel value = 0, 0.38 cd/m^2^), grey (pixel value = 128, 18.1 cd/m^2^), or white (pixel value = 255, 98.0 cd/m^2^). The luminance contrast of the line color was also manipulated. The contrast was either strong (75% of the maximum contrast, pixel value was 32 and 224, and luminance was 0.53 and 72.45 cd/m^2^, respectively) or weak (25% of maximum contrast, pixel value was 96 and 160, and luminance was 8.37 and 31.7 cd/m^2^). Each condition was presented eight times in a pseudorandom sequence, leading to 480 trials in total (2 shape × 3 background ×  2 contrast × 5 offset × 8 repetition).

### Results

**Figure 5.**
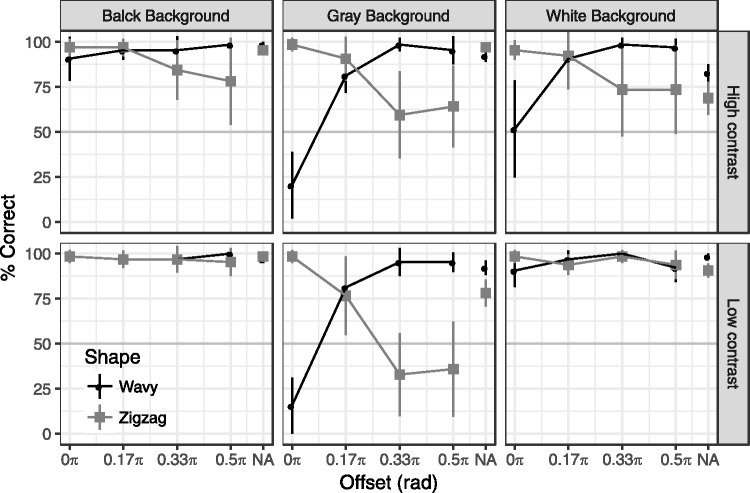
Results of Experiment 2. Error bars indicate 95% confidential intervals. See Figure 3 for more details.

Figure 5 shows the results of Experiment 2. We performed a four-way repeated measures ANOVA of shape, background, contrast, and offset. As the four-way interaction was significant, *F*(8,56) = 6.90, *p* < .01, $\eta_{\text{p}}^{2}$^ ^= 0.50, we tested the effects of offset for each condition. The effects of offset were significant in the all gray background conditions and white background conditions with the high contrast, *F*s(4, 28) > 3.88, *p*s < .05, η_p_^2 ^> 0.36. The significant bias from the wavy to zigzag line was observed at the offset 0π in both strong (95% CI = [0.02, 0.39]) and weak contrast (95% CI = [0.01, 0.31]) conditions with the gray background. No other condition showed significant bias. These results showed that the curvature blindness illusion takes place only when the background is gray. In this condition, the luminance contrast polarity of the wavy line was reversed against the background at the turning point. We also found that the contrast strength of black and white on the line has little effects. The detection of a zigzag line was difficult when the color changed around the middle of the turning point (offset = 0.33π, 0.50π).

## Experiment 3

Assuming the shadowing by a single light source, the change in luminance at the corner gives the impression of the change of surface orientation. Indeed, we can see depth for the patterns with offset 0π in Figures 2 and 4. Given the potential involvement of the depth perception, it would be reasonable that the surface is straight rather than curved since the luminance is constant along the segmented regions of lines. In other words, if a surface is curved, the shadowing will make a gradual change of luminance. Therefore, it is possible to argue that the curvature blindness illusion would arise from the inference of surface orientation from the depth percepts and shadowing. To test this possibility, in Experiment 3, we made the pattern wherein the contrast polarity is discontinuous but depth cue is not available (Figure 2, row 7, left). If the depth percepts are crucial, this pattern should not induce the curvature blindness illusion.

### Methods

Eight naïve university students different from those in Experiments 1 and 2 were recruited. The methods were identical with Experiment 1 except for the following. Figure 1 shows some examples of the visual stimuli. In Experiment 3, only the low curvature or angle stimuli were presented. We presented the black or white alternation conditions and the black segment conditions (Figure 1, second and third from bottom). The alternation condition was identical with Experiment 1. In the segment condition, black segments were placed on a white line instead of alternating white and black color. Each condition was presented 16 times in a pseudorandom sequence, leading to 480 trials in total (2 shape × 2 color change × 7 offset × 16 repetition).

### Results

**Figure 6.**
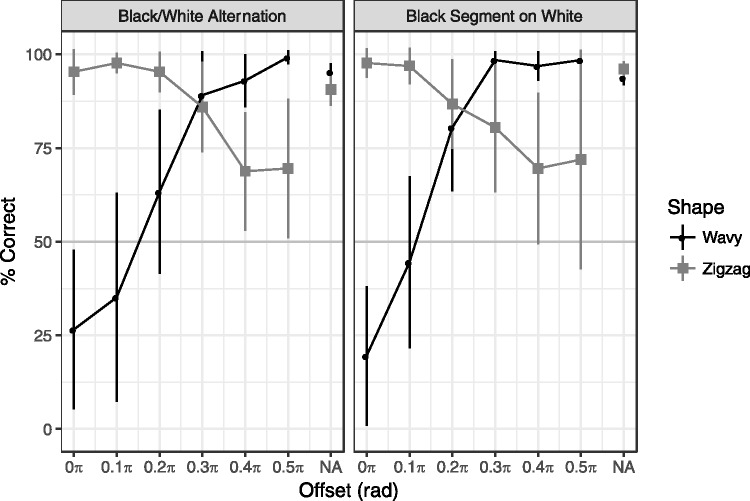
Results of Experiment 3. Error bars indicate 95% confidential intervals. See Figure 3 for more details.

Figure 6 shows the results of Experiment 3, which replicated Experiment 1. Furthermore, the black segments on the white line efficiently induced the curvature blindness illusion. A three-way repeated measures ANOVA of shape, color change, and offset revealed the significant three-way interaction, *F*(6, 42) = 2.74, *p* = .02, η_p_^2 ^= 0.28. The pattern was similar across the white or black alternation and black segments on white line, which was supported by no significant interactions in the two-way repeated measures ANOVAs of offset × color change; wavy shape condition: *F*(6, 42) = 1.97, *p* = .12, η_p_^2 ^= 0.22; zigzag shape condition: *F*(6, 42) = 1.41, *p* = .24, η_p_^2 ^= 0.17.

The significant bias from the wavy to zigzag line was observed in both black or white alternation (95% CI = [0.05, 0.48]) and black segments (95% CI = [0.01, 0.38]) with the offset = 0π. No other condition showed significant bias.

## General Discussion

The present study reported and explored a novel illusion wherein a wavy line is perceived as a zigzag line (Figure 1). The following were the identified prerequisites for this illusion to occur. First, the luminance contrast polarity of the wavy line needs to be reversed against the background at the turning points (Experiment 2). Second, the polarity can be reversed before and after the turning point or only at the turning point. In both cases, the illusion is efficiently induced (Experiment 3). Thus, the depth percepts would not be a crucial factor for this illusion. Third, the curve needs to be somewhat gentle; the right angle is too steep for the illusion to occur (Experiment 1).

This illusion has two implications. First, the percepts of a gentle curve (wavy line) are impaired by discontinuity of contrast polarity at the turning point, while the percepts of an obtuse corner are not; this implies that the mechanisms of the gentle curve and obtuse corner detection are separable in terms of contrast polarity. Second, the illusory percepts of an obtuse corner (zigzag line) appears and replaces the gentle curve, which indicates the imbalanced competition between these two percepts.

Some models propose that the end-stop cells play a role in curvature detection (Dobbins et al., 1987, 1989; Skottun, 1998). These models, however, efficiently work with relatively high curvature. Experiment 1 revealed that the high curvature is not impaired by the discontinuity of contrast polarity. Therefore, the curvature detection mechanism including end-stop cells are not susceptive to the discontinuity of contrast polarity and not relevant to the curvature blindness illusion.

The models of low-curvature detection presume combination of the outputs from orientation selective cells (Loffler, 2008; Poirier & Wilson, 2006; Wilson, 1985; Wilson & Richards, 1989). Although these models did not consider the contrast polarity of the orientation cells, other psychophysical studies demonstrated that the mechanisms of curvature detection are selective to the luminance contrast polarity (Bell, Gheorghiu, Hess, & Kingdom, 2011; Gheorghiu & Kingdom, 2006). The present study also implies that the low curvature detector would combine the local and neighboring orientation filters in a selective way depending on the contrast polarity of orientation filters. Taken together, the local orientation filters need to have the same contrast polarity to be combined to construct the low-curvature representation; otherwise, the low curvature is hardly detected.

The failure of the detection of the low curvature might be related to the impaired curvature detection by line segmentation (Watt, 1984, 1985). In a series of psychophysical studies, Watt and his colleagues demonstrated that the segmentation of a curved line by line intersections (Watt, 1985) or gaps (Watt, 1984) elevated the curvature detection threshold. In the present study, the change of luminance contrast resulted in the percepts of segmentation, which would disrupt the curvature detection. Note that, since the illusory effect was absent for the segmented lines in the high-curvature condition (Experiment 1) and in the white and black background condition (Experiment 2), the line segmentation might be necessary condition yet would not be sufficient condition.

The perception of obtuse corner was not impaired by the discontinuity of contrast polarity at the corner. Like a detection of curvature, the detection of corner is also modeled as the combination of orientation filters (Ito & Komatsu, 2004; Snippe & Koenderink, 1994). Unlike the low-curvature detector, however, the obtuse corner detector would combine the local orientation filters regardless of their contrast polarity.

Thus, even though low-curvature detection and obtuse corner detection would be achieved by combining the local and neighboring orientation filters, the visual system seems to have separable integration processes, intermediate representations, and detectors for low curvature and obtuse corner.

The illusion demonstrates that one exactly “sees” an illusory zigzag pattern against a physically wavy pattern. Even if the curvature and corner are detected in a separate process, impairment of the percepts of low curvature does not necessarily mean that the percepts of obtuse corner will appear. Then, how can the physically wavy lines be replaced by the perceptually zigzag lines? Here, we speculate the relation between the low-curvature detector and the obtuse corner detector.

The first assumption is that the visual input of low curvature would satisfy the requirement to trigger the obtuse corner detector, since there are surely two different orientations before and after the turning point of the low curvature. The second assumption is that the low-curvature detector combines the outputs from the local orientation filters with the same luminance contrast polarity, whereas the obtuse corner detector does not care of the contrast polarity of local orientation filters. The first assumption would be supported by the physiological studies that identified some cells responding both curves and angles (Hegdé & Van Essen, 2007). In a normal condition, however, we do not perceive an obtuse corner against a low curvature. This is because a low curvature detector, which was also triggered by the input of the low curvature, interferes with the output of the obtuse corner detector. Thus, the percepts of low curvature “win” there. On the other hand, in the curvature blindness illusion, the visual inputs of low curvature do not activate the low curvature detector because of the inverted contrast polarity across local orientation filters. However, the obtuse corner detectors still respond to the low-curvature inputs because the contrast polarity does not matter (second assumption). Then, there is no interference from the obtuse corner detector; thus, even if the input is a wavy line, the percepts of zigzag corner “win” without fighting.

This account of unbalanced competition would be consistent with a previous study on shape adaptation (Ito, 2012). This study demonstrated that the adaptation to a circle (i.e., low curvature) induces the afterimage of a hexagon (i.e., obtuse corner). They also reported that one (including me) notices that a circle deforms to a polygon during simple observation. These results indicate that the low-curvature detector and obtuse corner detector compete to each other. Furthermore, while some people reported that the adaptation to a hexagon induces the afterimage of a circle, the transformation from a circle to a hexagon seems to be more effective than the transformation in the opposite direction. These results also indicate that the rivalry between the low-curvature detector and obtuse corner detector are not balanced but that the corner detector is dominant. Taken together, it seems to be a robust effect that the percepts of obtuse corner appears when the percepts of low curvature are impaired by fatigue due to adaptation (Ito, 2012) or reversal of contrast polarity (curvature blindness illusion). Further investigation on the interaction between different detectors––rivalry, imbalance, and dominance––would be a promising way to draw a whole picture of contour and shape perception.

Finally, not directly related to the curvature blindness illusion, we note the puzzling effects of the obtuse corner detection. When the contrast polarity was reversed around the middle of two corners (offset = 0.33π and 0.5π), the corner detection was impaired in all experiments. Indeed, this pattern (see Figure 4, right column, row 4) gives an impression that the angle is less steep. See also the bottom-right (a white zigzag) and one above it (black segment on a white zigzag) in Figure 2. While there is no difference at the corner, the corner of the former pattern is easy to detect while the corner of the latter is rather difficult (Experiment 3, Figure 6). The reason is open, but global shape perception might have influenced the local obtuse angle detection (Kennedy, Orbach, & Loffler, 2006, 2008). Another interesting observation is that the perceived frequency of the waves is increased when the contrast polarity changes at the middle of two turning points (Figures 2 and 4, offset = 0.5π conditions). Further investigation of these unexpected effects will allow us to extend the understanding of the mechanisms of corner detection.
